# Management of Respiratory Syncytial Virus Bronchiolitis: 2015 Survey of Members of the European Society for Paediatric Infectious Diseases

**DOI:** 10.1155/2016/9139537

**Published:** 2016-10-20

**Authors:** Elliott J. Carande, Andrew J. Pollard, Simon B. Drysdale

**Affiliations:** Oxford Vaccine Group, Department of Paediatrics, University of Oxford and the NIHR Oxford Biomedical Research Centre, Level 2, Children's Hospital, Oxford OX3 9DU, UK

## Abstract

In 1995, the European Society for Paediatric Infectious Diseases (ESPID) carried out a survey of its members to assess the variation in management of respiratory syncytial virus (RSV) bronchiolitis. The aim of the current study was to carry out a similar survey 20 years later to assess how the management had changed. An electronic, structured, English language survey, based on the United Kingdom National Institute for Health and Care Excellence (NICE) bronchiolitis draft guideline, was sent to ESPID members in March 2015. Questions asked included information on treatment practices of infants with bronchiolitis and doctor demographics. We received responses from 135 doctors (14% of the ESPID members) who worked in 115 hospitals. 56% of the doctors used a written guideline to manage bronchiolitic infants. All doctors stated that they isolated individually or in cohorts all hospitalised bronchiolitis infants. The level of oxygen saturation suggested as an indication to administer supplemental oxygen varied between <89% and <95%. We found significant reductions in the use of ribavirin, bronchodilators, and corticosteroids from 1995 to 2015 (ribavirin 57% to 13%, *P* < 0.0001; bronchodilators 95% to 82%, *P* = 0.0024; corticosteroids 81% to 45%, *P* < 0.0001). Although variability in management remains high, encouragingly significantly fewer doctors are prescribing ribavirin, bronchodilators, and corticosteroids compared to 20 years ago.

## 1. Introduction

Respiratory syncytial virus (RSV) infects almost all children by two years of age. In the UK and other developed nations, between 1 and 4% of the entire birth cohort is hospitalised due to viral bronchiolitis each year, with a large degree of regional variability [1]. RSV is the cause of up to 80% of hospital admissions for viral bronchiolitis. Worldwide, after malaria, RSV is the organism causing the greatest mortality in postneonatal infants [2].

The management of RSV bronchiolitis (and that caused by other viruses) is purely supportive with supplemental oxygen and feeding support [3]. Ribavirin is a medication with anti-RSV properties which has been used widely previously; however, recent evidence suggests it should not be used in the vast majority of infants with RSV bronchiolitis due to its high cost, the difficulty of its administration to ventilated patients, potential adverse effects, and limited impact on outcomes [4]. There is also excellent evidence that other treatments such as bronchodilators, steroids, antibiotics, montelukast, hypertonic saline, and physiotherapy have no role in the vast majority of infants [5–11]. Investigations such as chest X-ray and blood gas are usually unhelpful and should be reserved for infants with severe or atypical disease [3, 12]. The monoclonal antibody palivizumab is available to prevent severe RSV disease; however, due to its extremely high cost, it is only used in very limited numbers of infants (e.g., those born extremely prematurely or those with chronic respiratory or cardiac conditions) in high resource settings [13]. There is no licensed RSV vaccine.

In 1995, the European Society for Paediatric Infectious Diseases (ESPID) carried out a survey of its members to assess the variation in management of RSV bronchiolitis in infants throughout Europe [14]. The aim of the current study was to carry out a similar survey 20 years later to assess how the management of RSV bronchiolitis has changed over time.

## 2. Methods

An electronic, structured, English language survey, based on the United Kingdom (UK) National Institute for Health and Care Excellence (NICE) bronchiolitis draft guideline (the full guideline was published in June 2015 [3]), was sent to members of ESPID (*n* = 970) in March 2015. The questions asked included information on treatment practices in infants with RSV bronchiolitis (including investigations, level of oxygen saturation requiring supplementation, and the use of steroids, bronchodilators, ribavirin, and other treatments) and demographics (seniority and specialty of the doctor completing the questionnaire and location and type of the hospital).

### 2.1. Definitions

In the 1995 survey, high-risk patients were defined as per the 1994 (23rd edition) American Academy of Pediatrics (AAP) Report of the Committee on Infectious Diseases (“Red Book”) and included infants with complicated congenital heart disease, bronchopulmonary dysplasia, cystic fibrosis, and other lung conditions; premature infants; children with immunodeficiency; recent transplant recipients; patients undergoing chemotherapy for malignancy; infants who are severely ill; and all patients mechanically ventilated for RSV infection [14].

In the 2015 survey, we categorised patients by disease severity rather than high-risk categories: any infant with RSV bronchiolitis, infants requiring hospitalisation, or infants requiring high dependency/intensive care.

### 2.2. Statistical Analysis

Proportions were compared using Fisher's exact test. Statistical analysis was carried out with IBM SPSS Statistics (version 22, New York, USA).

This study did not require ethical approval.

## 3. Results

We received responses from 135 doctors (14% of the ESPID members) who worked in 115 hospitals across the world. Of those, 109 members (12% of the ESPID members) from 95 hospitals fully completed the questionnaire (96 in Europe [17 from UK, 13 from Spain, 8 from Portugal, 7 from Switzerland and Netherlands, 6 from Finland, 5 from Germany and Greece, 4 from Romania, 3 from Italy, Poland, and Sweden, 2 from Austria, Belarus, and Slovenia, and 1 from Albania, Armenia, Belgium, Denmark, Iceland, Ireland, Latvia, Russia, and Ukraine], 6 in Asia, 2 in Africa, 2 in South America, 2 in Australasia, and 1 in North America). There were 83 (76%) consultants, 16 (15%) trainees, and 10 (9%) other grades. Of the 83 consultants, 45 (41%) were subspecialists in infectious diseases, 32 (29%) were general paediatricians, 2 (2%) were subspecialists in respiratory medicine, 2 (2%) were in paediatric intensive care, and 2 (2%) listed their specialty as “other.” 67 (61%) worked in university hospitals, 25 (23%) in district hospitals, 7 (6%) in community hospitals, and 10 (9%) in “other” settings. Only 61 (56%) doctors used a written guideline for the management of infants with bronchiolitis.

### 3.1. Viral Testing and Infant Isolation

72 (66%) doctors advised respiratory virus testing in all hospitalised bronchiolitic infants, 21 (19%) in only a subset of hospitalised bronchiolitic infants (e.g., only those with severe disease requiring HDU/PICU), and 8 (7%) in all infants with bronchiolitis presenting to the emergency department (ED) even if they were discharged home, and 8 (7%) doctors advised against taking respiratory samples from any infants. 50 (46%) doctors worked in hospitals that used point of care tests (POCTs) in the ED to test for RSV infection in infants being admitted to the hospital and 45 (41%) used POCTs to help decide which inpatients to isolate in inpatient wards. Ninety-three (85%) doctors sent respiratory samples to the microbiology/virology laboratory for testing. 57 (61% of those who sent samples to the laboratory) doctors responded that their hospital laboratory routinely tested samples for RSV, influenza, and other respiratory viruses, 25 (27%) only tested samples for RSV and influenza viruses, 8 (9%) only tested samples for RSV, 8 (9%) tested for “other” respiratory viruses, and 1 (1%) only tested for influenza viruses.

29 (27%) doctors reported that their hospital isolated infants with RSV infection separately from infants with bronchiolitis caused by other viruses, 25 (23%) doctors put all bronchiolitic infants requiring hospital admission into a cubicle/single room and did not create “bronchiolitis bays” (inpatient areas [“bays”] containing cots for more than one infant with a diagnosis of viral bronchiolitis), 22 (20%) isolated infants with the same virus together (i.e., separate bays for infants testing positive for RSV, influenza, human metapneumovirus, etc.), 14 (13%) created a “bronchiolitis bay” for all infants with bronchiolitis irrespective of the virus(es) the infants had, and 11 (10%) doctors had another unspecified infant isolation policy.

### 3.2. Investigations and Interventions

83 (76%) doctors suggested only carrying out a blood gas in hospitalised infants with severe bronchiolitis requiring HDU/PICU, 12 (11%) in all hospitalised infants with bronchiolitis, and 7 (6%) in all infants with bronchiolitis (including those not requiring hospitalisation) and 7 (7%) advised against doing blood gas routinely. 65 (60%) doctors suggested only performing a chest X-ray in hospitalised infants with severe bronchiolitis requiring HDU/PICU, 26 (23%) advised against routinely doing chest X-rays, 9 (8%) advised chest X-rays for all hospitalised infants (even if not requiring HDU/PICU) with bronchiolitis, and 9 (8%) advised chest X-rays for all infants with bronchiolitis (including those not requiring hospitalisation). The level of oxygen saturation suggested as an indication to administer supplemental oxygen varied between <89% and <95% (Table 1).

90 (83%) doctors reported that they administered supplemental oxygen on general paediatric wards (excluding HDU/PICU) using low flow (≤2 L/min) nasal cannulae, 55 (50%) used face masks, 41 (38%) used humidified high flow oxygen therapy (e.g., “Optiflow,” “Vapotherm,” and “Airvo”), 25 (23%) used continuous positive airway pressure (CPAP), 17 (16%) used a headbox, and 9 (8%) used an incubator. The totals add up to more than 100% as most doctors responded that they used more than one method to administer supplemental oxygen.

The interventions used to manage infants with bronchiolitis varied considerably (Table 2).

When comparing the use of bronchodilators (via inhaler or nebuliser), corticosteroids (oral, via inhaler or nebuliser), or ribavirin in any patient (combining all “risk/severity groups”) and those doctors not using the medication in any patient between 1995 and 2015, we found significant reductions in the use of each of the three medications from 1995 to 2015 (ribavirin 57% to 13%, *P* < 0.0001; bronchodilators 95% to 82%, *P* = 0.0024; corticosteroids 81% to 45%, *P* < 0.0001) (Table 3).

### 3.3. Written Advice

Overall, 43 (39%) doctors gave written advice to parents of bronchiolitic infants: 24 (22%) if discharged from the ED or the inpatient ward, 12 (11%) if discharged from ED (and not if discharged from the inpatient ward), and 7 (6%) if discharged from the inpatient ward (and not if discharged from the ED).

## 4. Discussion

We have demonstrated a wide variation in the management of infants with RSV bronchiolitis by members of ESPID, predominantly in Europe. In 2015, fewer doctors prescribed bronchodilators, corticosteroids, and ribavirin than in 1995, but many infants still routinely received inappropriate medications and underwent unnecessary investigations despite a lack of evidence for their efficacy.

The 1995 ESPID poll [14] focussed on the use of bronchodilators, corticosteroids, and ribavirin. When comparing the use of those medications between the two surveys, we have found a statistically significant reduction in the use of each of those medications in line with mounting evidence that they do not improve patient outcomes (i.e., have limited efficacy) and are associated with side effects [4–6]. In addition, many national guidelines have been published in the interim between the two surveys advising against the use of those medications in almost all infants [3, 12, 15, 16]. Encouragingly, we noted that of the doctors who did advise those medications in the 2015 survey they did so primarily for only selected high-risk infants/severely unwell infants who may receive some benefit. Despite an overall reduction in the use of those medications between the time points, there was still a high degree of variability in overall RSV bronchiolitis management including investigations undertaken, requirement for starting supplemental oxygen, other medications' use, and other interventions in 2015. This is perhaps unsurprising, given that only 56% of the doctors followed a written guideline.

Our study has several strengths and a number of limitations. We have surveyed a large number of ESPID members covering a range of countries, predominantly within Europe, and received a similar number of completed responses to the 1995 survey (88 in 1995 and 109 in 2015). However, only 12% of the total number of members of ESPID completed the 2015 survey potentially biasing the results as the responses we received may not have been representative of all ESPID members. The 2015 survey included different grades of doctors (trainees and consultants) working in a variety of different hospital settings (university hospitals to community hospitals) and specialties (infection specialists and general paediatricians) suggesting that the data are generalizable to other hospitals. There were differences in the questions asked in the two surveys and how infants were categorised, as high-risk or by increased severity of illness, which may have influenced how the respondents completed the questionnaire and could have affected the results. We feel, however, that this is unlikely to have significantly influenced the results of the study and the overall results are in keeping with other studies demonstrating an improvement in care with time [12].

## 5. Conclusion

In summary, there remains a wide variation in the management of RSV bronchiolitis in infants by members of ESPID. Although variability in management remains high, encouragingly significantly fewer doctors are prescribing ribavirin, bronchodilators, and corticosteroids compared to 20 years ago. Ongoing education is required to ensure this trend continues.

## Figures and Tables

**Table 1:** The number (%) of doctors who administer supplemental oxygen at the given level of oxygen saturation.

Oxygen saturation	Number (%) of doctors
<89%	1 (1%)
<90%	23 (21%)
<91%	2 (2%)
<92%	52 (48%)
<93%	10 (9%)
<94%	9 (8%)
<95%	8 (7%)
Other	4 (4%)
Total	109 (100%)

**Table 2:** Number (%) of doctors advising various interventions to manage infants with RSV bronchiolitis in various settings.

	Bronchodilators via inhaler	Bronchodilators via nebuliser	Nebulised adrenaline	Steroids via inhaler	Steroids via nebuliser	Oral steroids	Heliox	Montelukast	Nebulised hypertonic saline	Antibiotics	Ribavirin	RSV intravenous immunoglobulin	Palivizumab	Chest physiotherapy	Nasal suctioning	Naso-/orogastric feeding	Intravenous fluids
Most patients with bronchiolitis (whether admitted to an inpatient ward or discharged home)	18 (17%)	14 (13%)	9 (8%)	4 (4%)	4 (4%)	2 (2%)	0 (0%)	1 (1%)	20 (18%)	3 (3%)	1 (1%)	0 (0%)	0 (0%)	4 (4%)	19 (17%)	1 (1%)	6 (6%)
Most patients with bronchiolitis admitted to an inpatient ward	6 (6%)	25 (23%)	14 (13%)	1 (1%)	3 (3%)	2 (2%)	1 (1%)	0 (0%)	29 (27%)	4 (4%)	0 (0%)	0 (0%)	2 (2%)	6 (6%)	43 (39%)	21 (19%)	18 (17%)
Most patients with severe bronchiolitis (i.e., requiring high dependency/intensive care)	5 (5%)	17 (16%)	27 (25%)	9 (8%)	11 (10%)	19 (17%)	10 (9%)	6 (6%)	13 (12%)	22 (20%)	5 (5%)	5 (5%)	3 (3%)	16 (15%)	20 (18%)	53 (49%)	62 (57%)
Other indications in infants with bronchiolitis	20 (18%)	18 (17%)	11 (10%)	11 (10%)	3 (3%)	11 (10%)	1 (1%)	4 (4%)	12 (11%)	26 (24%)	8 (7%)	2 (2%)	6 (6%)	11 (10%)	6 (6%)	12 (11%)	17 (16%)
Not administered to any patient with bronchiolitis	60 (55%)	35 (32%)	48 (44%)	84 (77%)	88 (81%)	75 (69%)	97 (89%)	98 (90%)	35 (32%)	54 (50%)	95 (87%)	102 (94%)	98 (90%)	72 (66%)	21 (19%)	22 (20%)	6 (6%)

**Table 3:** Comparison of the use of ribavirin, bronchodilators, and corticosteroids in 1995 with 2015.

Year	Ribavirin			Bronchodilators			Corticosteroids		
	1995	2015	*P* value	1995	2015	*P* value	1995	2015	*P* value
Number	88	109		88	109		88	109	
All patients	0 (0%)	1 (1%)	0.55	54 (61%)	28 (26%)	<0.0001	10 (11%)	7 (6%)	0.17
All high-risk patients	16 (18%)	0 (0%)	<0.0001	15 (17%)	22 (20%)	0.35	23 (26%)	4 (4%)	<0.0001
Selected high-risk patients^*∗*^	34 (39%)	13 (12%)	<0.0001	15 (17%)	39 (36%)	0.0025	38 (43%)	38 (35%)	0.15
Total use (above groups combined)	50 (57%)	14 (13%)	<0.0001	84 (95%)	89 (82%)	0.0024	71 (80%)	49 (45%)	<0.0001

^*∗*^“Selected high-risk patients” means the medication is only advised for some high-risk patients (rather than to every high-risk patient). We did not investigate which subgroups of high-risk patients would receive each of the medications.
